# CD93 Signaling via Rho Proteins Drives Cytoskeletal Remodeling in Spreading Endothelial Cells

**DOI:** 10.3390/ijms222212417

**Published:** 2021-11-17

**Authors:** Stefano Barbera, Luisa Raucci, Roberta Lugano, Gian Marco Tosi, Anna Dimberg, Annalisa Santucci, Federico Galvagni, Maurizio Orlandini

**Affiliations:** 1 Department of Biotechnology, Chemistry and Pharmacy, University of Siena, 53100 Siena, Italy; stefano.barbera@student.unisi.it (S.B.); luisa.raucci@unisi.it (L.R.); annalisa.santucci@unisi.it (A.S.); federico.galvagni@unisi.it (F.G.); 2 Department of Immunology, Genetics and Pathology, Science for Life Laboratory, Uppsala University, Rudbeck Laboratory, SE-75185 Uppsala, Sweden; roberta.lugano@igp.uu.se (R.L.); Anna.Dimberg@igp.uu.se (A.D.); 3 Ophthalmology Unit, Department of Medicine, Surgery and Neuroscience, University of Siena, 53100 Siena, Italy; gianmarco.tosi@unisi.it

**Keywords:** Src, Cbl, Crk, Rac1, Cdc42, RhoA

## Abstract

During angiogenesis, cell adhesion molecules expressed on the endothelial cell surface promote the growth and survival of newly forming vessels. Hence, elucidation of the signaling pathways activated by cell-to-matrix adhesion may assist in the discovery of new targets to be used in antiangiogenic therapy. In proliferating endothelial cells, the single-pass transmembrane glycoprotein CD93 has recently emerged as an important endothelial cell adhesion molecule regulating vascular maturation. In this study, we unveil a signaling pathway triggered by CD93 that regulates actin cytoskeletal dynamics responsible of endothelial cell adhesion. We show that the Src-dependent phosphorylation of CD93 and the adaptor protein Cbl leads to the recruitment of Crk, which works as a downstream integrator in the CD93-mediated signaling. Moreover, confocal microscopy analysis of FRET-based biosensors shows that CD93 drives the coordinated activation of Rac1 and RhoA at the cell edge of spreading cells, thus promoting the establishment of cell polarity and adhesion required for cell motility.

## 1. Introduction

Migration of endothelial cells (ECs) is essential for the outgrowth of new blood vessels both in physiological and pathological conditions. ECs of the vascular bed are usually stable and quiescent; however, when the environment surrounding the endothelium becomes hypoxic, the ECs switch to a proliferative and migratory state, leading to vascular remodeling [1]. In migrating cells, the integration of several cues, largely provided by adhesion to the substrate, is critical to activate intracellular signals that bias cytoskeletal remodeling, cell shape, and polarity [2]. Therefore, uncovering the molecular mechanisms that underpin the regulation of EC adhesion could allow a better understanding of both physiological and pathological angiogenesis.

In addition to integrins, stimulated endothelia express alternative adhesive proteins that can bind extracellular matrix (ECM) components and activate signaling pathways during cell motility [3]. Among these proteins, the C-type lectin transmembrane protein CD93, predominantly expressed in ECs, has been extensively proven to play important roles in vascular biology [4,5,6]. CD93 is upregulated in the endothelium of different types of vascularized tumors where it promotes its proangiogenic activities regulating cell adhesion and migration through interaction with different proteins [7,8]. Indeed, CD93 interacts with β-dystroglycan, which is a laminin-binding protein found to be upregulated in ECs of growing blood vessels within malignant tumors [9,10]. Moreover, the binding of CD93 to Multimerin-2, an endothelial-specific ECM protein [11], contributes to progression of the neovascular form of age-related macular degeneration, while in gliomas, it promotes β1 integrin activation and the fibrillar organization of fibronectin, increasing the motility of proliferating ECs [7,8,12]. Furthermore, the pathway activated by the interaction between CD93 and insulin-like growth factor binding protein 7 (IGFBP7), an ECM protein upregulated in tumor blood vessels, contributes to abnormal tumor vasculature [13]. As a membrane receptor, CD93 mediates the transduction of signals from the extracellular milieu into the cell. Consistently, the cytoplasmic domain of CD93 interacts with different proteins and is essential for the endosomal trafficking of CD93 to the leading edge of migrating ECs [14,15]. In spreading cells, the Src kinase phosphorylates the cytotail of CD93 on two tyrosine residues, which generate consensus motifs for the binding of the adaptor protein c-Cbl that drives the regulation of in vitro angiogenesis [9]. In migrating cells, the phosphorylation of Cbl on tyrosine 774 triggers a signaling cascade that leads to the activation of Rho-proteins and remodeling of the cytoskeleton [16]. However, although the critical role played by CD93 in mediating cellular responses to extracellular cues is quite clear, the transduction pathway triggered by CD93 during EC adhesion has not yet been fully elucidated.

In the present work, we analyze the downstream signaling pathway regulated by CD93 in primary spreading ECs. We show that CD93-dependent Src activation, together with the recruitment of phosphorylated Cbl on tyrosine 774, triggers the activation of small GTPases at the cell edge of spreading cells, thus promoting cytoskeletal reorganization required for proper EC adhesion.

## 2. Results

### 2.1. CD93 Modulates Activation and Localization of Src Kinase

**Figure 1:**
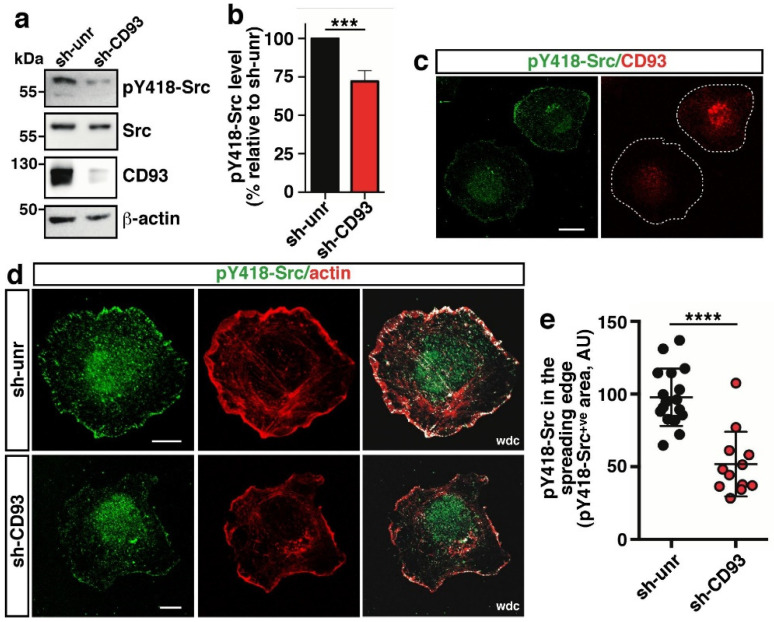
CD93 regulates the activation of Src in adhering ECs. HUVECs were transduced with lentiviral particles expressing unrelated (sh-unr) or CD93 (sh-CD93) shRNAs. (**a**) Total cell extracts from early spreading transduced HUVECs were analyzed by Western blotting with antibodies against pY418-Src, Src, and CD93. Anti-β-actin antibodies were used to confirm equal loading. (**b**) Quantitative analysis of pY418-Src levels from independent experiments performed as in (**a**). Values, normalized to total Src and β-actin protein levels, represent the percentage of Src phosphorylation in CD93-silenced cells (sh-CD93) relative to control (sh-unr). *** *p* < 0.001; paired *t*-test. (**c**) Transduced HUVECs were detached from the plate by a non-enzymatic method, resuspended in complete medium, mixed, reseeded, and fixed at the early phases of spreading. Cells were stained using antibodies against pY418-Src and CD93. White dashed lines indicate cell boundaries. Scale bar, 20 μm. (**d**) Representative images of immunofluorescence analysis of transduced ECs in the early phase of adhesion. Cells were stained using phalloidin and antibodies against pY418-Src. White dot colocalization (wdc) images between pY418-Src and F-actin are shown. Scale bar, 10 μm. (**e**) Quantification of cellular pY418-Src in the spreading border area (5 μm from the cell edge) of control cells (*n* = 17, sh-unr) and CD93-silenced cells (*n* = 12, sh-CD93). Data are presented as a scatter plot, and the fluorescence intensity of the pY418-Src^+ve^ signal per area is reported as arbitrary units (AU). **** *p* < 0.0001; Mann–Whitney test.

We have previously shown that in ECs adhering to the substrate, Src-dependent phosphorylation of the CD93 cytotail on tyrosines 628 and 644 generates two consensus sequences for the binding of the adaptor protein Cbl [9]. While these findings outlined the involvement of CD93 in the activation of a signaling pathway regulating cell adhesion, CD93-dependent regulation of Src activity was unclear prior to this investigation. To address this issue, human umbilical vein ECs (HUVECs) were transduced with a lentiviral construct carrying a CD93 specific shRNA sequence, detached from the plate, and the reseeded sparse cells, after an adhesion period assessed as early spreading phase [17], were analyzed by immunoblotting. Analyses of cell lysates from early spreading HUVECs showed decreased levels of Src phosphorylated at tyrosine 418, which is a protein modification that is closely correlated with kinase activity, in CD93-silenced cells compared to control ECs (Figure 1a, quantified in Figure 1b). These observations were further substantiated by immunofluorescence analyses showing that active Src was strongly decreased at the cell border of CD93-silenced ECs compared to ECs transduced with a lentivirus expressing an unrelated shRNA (Figure 1c). Furthermore, immunofluorescence analysis, showing the colocalization of F-actin with active Src in early adhering ECs, highlighted that the activation of Src was significantly decreased at the cell edge of CD93-depleted ECs in comparison to control cells (Figure 1d, quantified in Figure 1e). These data, together with previous findings showing that CD93 silencing strongly affected adhesion and actin cytoskeletal organization of ECs [5,7], suggest that CD93 drives Src activation and its recruitment to the cell edge to allow proper EC adhesion.

### 2.2. CD93 Controls Phosphorylation and Cellular Localization of Cbl

**Figure 2:**
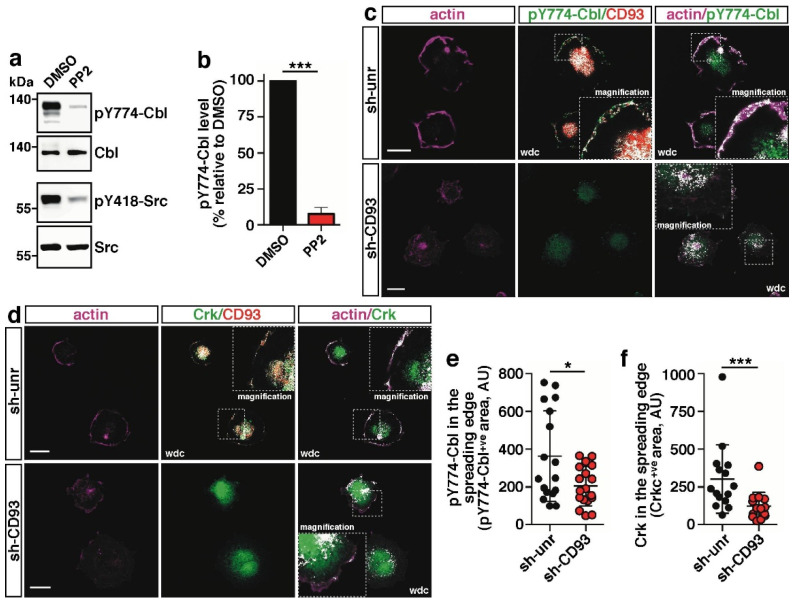
CD93 triggers the pY774-Cbl-dependent signaling pathway in spreading ECs. (**a**) Cell lysates from early spreading HUVECs pretreated for 30 min with vehicle alone (DMSO) or 10 μM PP2 were analyzed by Western blotting using antibodies against pY774-Cbl and pY418-Src to confirm PP2 activity. Equal loading was confirmed by using anti-Cbl and anti-Src antibodies. (**b**) Quantification of pY774-Cbl phosphorylation levels from independent experiments performed as in (**a**). Values, normalized to Cbl protein levels, represent the percentage of pY774-Cbl levels relative to vehicle-treated cells (DMSO). *** *p* < 0.001; paired *t*-test. (**c**,**d**) HUVECs were transduced with lentiviral particles expressing unrelated (sh-unr) or CD93 shRNA (sh-CD93). Cells were detached from the plate, resuspended in complete growth medium, plated on the substrate, and fixed at early phases of spreading. Cells were analyzed by immunofluorescence using phalloidin, anti-CD93 antibody, and antibodies against pY774-Cbl (**c**) and Crk (**d**). White dot colocalization (wdc) images are shown as indicated. In the wdc pictures, magnification of the squared areas is shown (2.5×). Scale bars, 20 μm. (**e**,**f**) Quantification of cellular pY774-Cbl (**e**) and Crk (**f**) in the spreading border area (5 μm from the cell edge) of HUVECs transduced and treated as in (**c**) (*n* = 14 cells for sh-unr and *n* = 16 cells for sh-CD93) and (**d**) (*n* = 15 cells for sh-unr and *n* = 14 cells for sh-CD93). Data are presented as scatter plots and the fluorescence intensity of the pY774-Cbl^+ve^ and Crk^+ve^ signals per area is reported as arbitrary units (AU). * *p* < 0.05; Student *t*-test (**e**). *** *p* < 0.001; Mann–Whitney test (**f**).

We have previously shown that the CD93-dependent phosphorylation of Cbl on tyrosine 774 is an important event in promoting EC adhesion [9]; however, it was unclear whether this regulation was also dependent on Src activation. To explore this possibility, we investigated the effect of PP2, a selective Src family kinase (SFK) inhibitor, on Cbl phosphorylation. Western blot analysis of cell lysates from spreading HUVECs revealed that the treatment with PP2 strongly impaired the activation of Src kinase and importantly the phosphorylation of Cbl on tyrosine 774, suggesting that this specific adaptor modification is SFK-dependent in ECs flattening out on the surface (Figure 2a, quantified in Figure 2b). Since in the migration front of ECs, the binding of pY774-Cbl to CD93 triggers a signaling cascade regulating cell dynamics [16], we asked whether this signaling pathway was also activated during cell spreading. To address this issue, we silenced CD93 in HUVECs and analyzed by immunofluorescence the subcellular localization of previously identified CD93-dependent signaling players [16]. Consistent with our previous results on EC migration [16], while CD93 colocalized with pY774-Cbl and the downstream modulator Crk at the cell edge (Figure 2c,d, sh-unr), the knockdown of CD93 dramatically impaired the localization of pY774-Cbl and Crk along the cell edge of ECs adhering to the substrate (Figure 2c,d, sh-CD93). These findings were further corroborated by quantitative analyses of pY774-Cbl and Crk at the cell edge of spreading cells (Figure 2e,f), providing further evidence of the key role played by CD93 in the regulation of the signaling pathway that controls EC adhesion and migration.

### 2.3. CD93 Regulates Activity of Rho-Proteins along the Cell Edge of Spreading ECs

**Figure 3:**
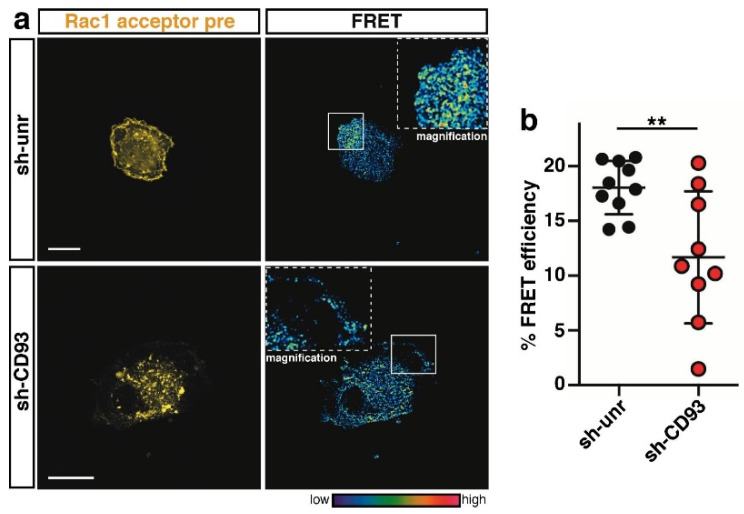
CD93 signaling activates Rac1 at the cell edge of spreading cells. (**a**) FRET analysis on control (sh-unr) or CD93-silenced (sh-CD93) HUVECs transduced with a lentiviral construct expressing the Rac1 biosensor. Cells were fixed at the early phase of adhesion to the ECM. Representative confocal images of transduced cells before photobleaching (acceptor pre) are shown. Rectangles indicate the photobleached cell area. Magnifications of the photobleached area are shown (2.5×). The colored scale represents the color range of FRET efficiency. Scale bars, 20 µm. (**b**) Plot showing the fluorescence increase (% FRET efficiency) upon photobleaching at the cell edge of early spreading ECs (*n* = 10 cells for sh-unr and *n* = 9 cells for sh-CD93). Data are presented as scatter plot. ** *p* < 0.01; Student *t*-test.

Since in motile ECs, the signaling triggered by CD93 converges to the activation and modulation of the Rho GTPase signaling pathways [16], we questioned whether the Rho-proteins Rac1, RhoA, and Cdc42 were CD93-dependent regulators of plasma membrane dynamics also during the early events of EC adhesion to the ECM. To address this possibility, control or CD93-silenced ECs were transduced with lentivirus constructs expressing biosensors that produce localized FRET signals, revealing the amount and location of Rac1, Cdc42, or RhoA activity [18,19,20]. Quantitative FRET analysis on the early spreading cells unveiled a statistically significant decrease in Rac1 activity at the cell edge of CD93-silenced ECs compared to control cells (Figure 3a, quantified in Figure 3b), suggesting that Rac1 is a downstream effector of the CD93 signaling pathway. 

**Figure 4:**
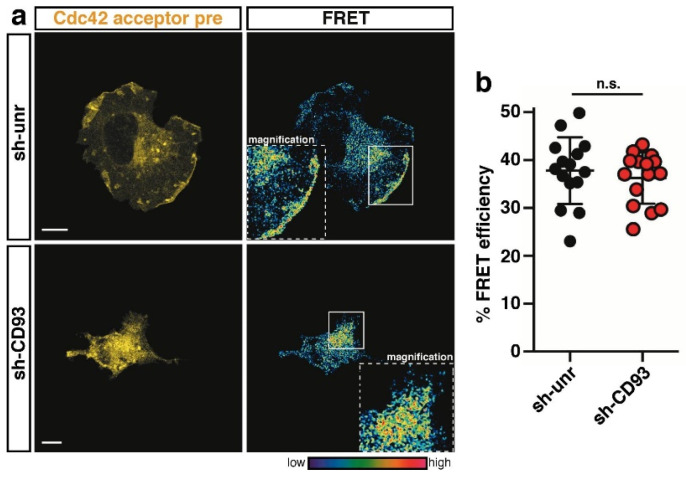
Cdc42 is not modulated by CD93 at the cell edge of adhering ECs. (**a**) FRET analysis on control (sh-unr) or CD93-silenced (sh-CD93) HUVECs transduced with a lentiviral construct expressing the Cdc42 biosensor. Cells were fixed at the early phase of adhesion to the ECM. Representative confocal images of transduced cells before photobleaching (acceptor pre) are shown. Rectangles indicate the photobleached cell area. Magnifications of the photobleached area are shown (1.6× sh-unr; 2.5× sh-CD93). The colored scale represents the color range of FRET efficiency. Scale bars, 10 µm. (**b**) Graph showing the fluorescence increase (% FRET efficiency) upon photobleaching at the cell edge of early spreading ECs (*n* = 15 cells for sh-unr and *n* = 15 cells for sh-CD93). Data are presented as scatter plot. n.s., not significant; Student *t*-test.

**Figure 5:**
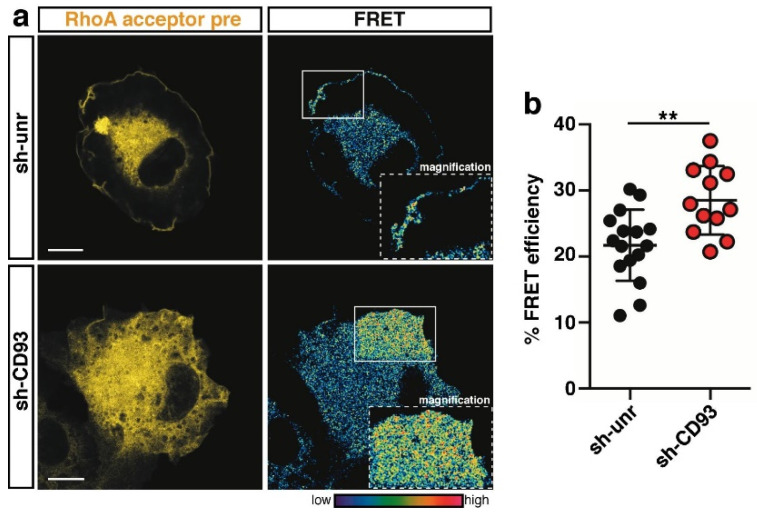
CD93 modulates RhoA activation at the cell edge of spreading ECs. (**a**) FRET analysis on control (sh-unr) or CD93-silenced (sh-CD93) HUVECs transduced with a lentiviral construct expressing the RhoA biosensor. Cells were fixed at the early phase of adhesion to the ECM. Representative confocal images of transduced cells before photobleaching (acceptor pre) are shown. Rectangles indicate the photobleached cell area. Magnifications of the photobleached area are shown (1.8× sh-unr; 1.5× sh-CD93). The colored scale represents the color range of FRET efficiency. Scale bars, 10 µm. (**b**) Plot showing the fluorescence increase (% FRET efficiency) upon photobleaching at the cell edge of early spreading ECs (*n* = 16 cells for sh-unr and *n* = 12 cells for sh-CD93). Data are presented as scatter plot. ** *p* < 0.01; Student *t*-test.

Consistent with its role played in the establishment of epithelial apicobasal polarity [21], Cdc42 was strongly activated at the cell edge of control spreading cells (Figure 4a, sh-unr); however, in contrast to Rac1 regulation, Cdc42 activation did not decrease in ECs lacking CD93 (Figure 4a, quantified in Figure 4b), suggesting that in the early phase of EC adhesion, the signaling pathway activated by CD93 does not affect Cdc42 activity. Importantly and in accordance with previous data on mouse fibroblasts [22], RhoA activity was concentrated in a narrow band at the cell edge of control cells (Figure 5a, sh-unr), whereas the lack of CD93 induced a statistically significant increase in RhoA activation at the cell edge of spreading ECs (Figure 5a, quantified in Figure 5b). Collectively, these results suggest that CD93, by modulating the activation of Rac1 and RhoA, controls the actin cytoskeleton dynamics necessary for a coordinated adhesion of ECs to the ECM.

## 3. Discussion

The adhesion of ECs to the ECM plays a critical role in modulating essential cellular functions such as migration, proliferation, and survival, via the activation of a complex network of intracellular signaling pathways. Hence, elucidating the signaling pathways that manage EC adhesion to the ECM may help to understand the physiology of angiogenesis-dependent diseases, offering the opportunity to unveil novel antiangiogenic targets. In the present work, we show that, similarly to what has been observed in migrating ECs [16], the transmembrane protein CD93 activates a signaling cascade involving Rac1 and RhoA small GTPases to control cytoskeletal movements responsible for EC spreading. This appears particularly interesting in view of the fact that, differently from quiescent blood vessels, CD93 is mainly expressed in ECs of growing new blood vessels in different types of neovascular pathologies [5,7,23].

Integrins are transmembrane proteins essential in mediating cellular responses downstream of ECM engagement [24]. Importantly, it has been shown that the binding of CD93 to the ECM promotes β1 integrin activation and activated integrins regulate Src activation [8,25], which is the protein kinase involved in the phosphorylation of the CD93 cytotail [9]. Consistent with these observations, we demonstrate that in spreading ECs, the activation and localization of Src on the cell edge depends on CD93 expression, suggesting that CD93 itself promotes the phosphorylation of its cytoplasmic domain via an integrin/Src signaling axis, which generates consensus sequences for the binding of the adaptor protein Cbl [9]. Of note, we show that the phosphorylation of Cbl on tyrosine 774 is also dependent on SFK activity, suggesting that the activation of Rho GTPases in adhesion signaling involves extensive crosstalk between integrins and Src kinases [26].

Adaptor proteins are crucial components of the signaling pathways and link activated receptors to specific downstream signals. We have previously shown that the CD93-dependent phosphorylation of Cbl on tyrosine 774, a protein modification associated with actin remodeling and enhancement of cell adhesion [27], is essential to promote EC ahesion [9]. Accordingly, we show that CD93 drives the reorganization of the actin cytoskeleton by controlling the proper localization of pY774-Cbl and its binding partner Crk on the cell edge of spreading ECs and by transducing intracellular signals similar to those observed in motile ECs [16], thus reinforcing the notion that cell adhesion and migration are tightly connected processes [28].

Rac1 activation and RhoA inhibition are specific characteristics of the early cell adhesion phase [17]. The use of FRET-based biosensors allowed us to investigate the activation of the Rho proteins in early spreading ECs, demonstrating a clear role for CD93 in the regulation of the spatially modulated activity of these molecules. It has been shown that Rac1 and RhoA are mutually inhibitory, and higher RhoA activity is localized in a narrow band at the cell border of migrating cells [16,22]. Consistent with these requirements, we show that RhoA activity exhibits the same sharp localization at the cell edge of control spreading ECs, but the loss of CD93 associates with a broadened cell area with increased RhoA activity and a consequent decreased of Rac1 activation at the cell edge, suggesting that the crosstalk between Rac1 and RhoA is crucial for the regulation of early adhesion events in ECs. Interestingly, elevated RhoA activity may suppress Rac1 activity and lamellipodium formation both via the activation of FilGAP, which is a Rac GTPase-activating protein, and via the restriction of the interaction between Rac1 and its guanine nucleotide exchange factor (GEF) β-Pix [29,30]. However, since the fine balance that exists between the opposing activities of Rac1 and RhoA is dictated by the spatiotemporal activation of several specific GAPs and GEFs, more work is required to understand how Rho-proteins signal to control specific phases of adhesion and spreading in ECs [17].

Angiogenesis is largely regulated by a finely tuned integration of external cues, which are integrated by ECs. Although speculative, we propose a molecular mechanism of how ECs attach and spread on ECM. When cells adhere on the substrate, they derive spatial cues from integrin signaling, which induces Src activation. At the same time, CD93 interacts with the ECM and promotes integrin activation, which leads to Src-dependent phosphorylation of the CD93 cytoplasmic domain. Phosphorylated CD93 recruits Cbl, which in turn undergoes SFK-dependent phosphorylation at tyrosine 774, generating a binding site for Crk and a signaling cascade for the reorganization of the actin cytoskeleton by modulating the Rho GTPases activity. In conclusion, this study sheds light into the understanding of the intricate molecular pathways activated by the microenvironment and translated by CD93 into actin dynamics during the adhesion process. 

## 4. Materials and Methods

### 4.1. DNA Constructs and RNA Interference

The following plasmids were purchased from Addgene (Watertown, MA, USA): a lentiviral negative control vector containing scrambled shRNA (#1864, sh-unr) [31]; Rac1 generation fluorescence resonance energy transfer (FRET) biosensor for lentivirus production (#66111, pLenti-Rac1-2G) [32]; FRET-based biosensor reporting on Cdc42 activation (#68813, pLenti-Cdc42-2G) [33]; RhoA second-generation FRET biosensor for lentivirus production (#40179, pLenti-RhoA-2G) [34].

shRNA-mediated knockdown of CD93 was performed as previously outlined [4] by using a pLKO.1 retroviral vector from the Mission shRNA Library (Merck KGaA, Darmstadt, Germany), which expresses a shRNA (clone TRCN0000029085) specific for the silencing of the human protein. Recombinant lentiviral particles were produced and used for infection experiments as earlier described [35].

### 4.2. Cell Culture

HUVECs from single donors were purchased from PromoCell (Heidelberg, Germany) and grown on gelatin-coated plates in Endothelial Cell Basal Medium (EBM-MV2) with supplements (PromoCell) as previously described [35]. For lentivirus production, human Lenti-X 293T cells (Takara Bio Inc, Kusatsu, Japan) were grown in DMEM containing 10% FBS and 1 mM sodium butyrate (Merck KGaA), which increases the viral titer [36]. To detach cells from the culture plate by the non-enzymatic method, growing cells were washed thrice with PBS, incubated with Cell Dissociation Solution Non-enzymatic (Merck KGaA) for 5 min at 37 °C, collected in M199 medium supplemented with 10% FBS, centrifuged, and resuspended in fresh culture medium.

### 4.3. Antibodies and Reagents

The following primary antibodies were used: mouse monoclonal anti-CD93 (clone 4E1) [4]; rabbit anti-phospho-Cbl(Tyr774) (04-334) and mouse anti-β-actin (A2228, Merck KGaA); mouse anti-Cbl (sc-1651) and normal mouse IgG (sc-2025, Santa Cruz Biotechnology, Dallas, TX, USA); rabbit anti-Src (32G6) and rabbit anti-phospho-Src(Tyr416) (D49G4, Cell Signaling Technology, Danvers, MA, USA). 

The Src family tyrosine kinase inhibitor PP2 was purchased from Merck KGaA. 

### 4.4. Western Blotting Analyses

Immunoblotting experiments were performed as previously described [37]. To compare protein levels of different samples, densitometric analysis was performed using the gel analyzer tool of ImageJ2.

### 4.5. Immunofluorescent and FRET Analyses

Acquisition of fluorescent images and FRET experiments were carried out using a Leica TCS SP5 AOBS confocal laser-scanning microscope, following the TCS SP5 and FRET AB manufacturer’s software. A Leica HC PL APO 63x/1.40 Oil CS2 objective was used for the acquisition of cell images and FRET analyses. A diode laser and argon laser were used to excite fluorochromes and fluorescent molecules at the optimal wavelength ranging from 405 to 647 nm, and images (1024 × 1024 pixel resolution) were acquired at a scan speed of 400 Hz image lines/sec. Confocal scanner configuration was set as follows: pinhole at 1.0 Airy diameter and line averaging function at 4.

For immunofluorescent staining, cells were fixed in 3% paraformaldehyde and treated as previously described [35]. The secondary appropriate antibodies were conjugated with Alexa Fluor-488 or -568 (Thermo Fisher Scientific, Waltham, MA, USA). F-actin staining was performed with Alexa Fluor-647 phalloidin (Thermo Fisher Scientific). To show colocalization events by white dots, images were generated using ImageJ2 and the Colocalization plug-in. To quantify protein levels at the cell border, an area of 5 µm distance from the cell edge was chosen, and images were analyzed using ImageJ2.

FRET acceptor photobleaching (apFRET) analyses were carried out on control or CD93-silenced HUVECs transduced with genetically encoded biosensors expressing RhoGTPases Rac1 (pLenti-Rac1-2G), Cdc42 (pLenti-Cdc42-2G), or RhoA (pLenti-RhoA-2G). Transduced cells were detached from the plate, resuspended in complete growth medium, plated on the substrate, fixed at early phases of spreading (from 30 to 40 min), and analyzed. Acquisition parameters were modified only in the pinhole diameter at 2.0 Airy units. Argon laser lines 458 nm and 514 nm were used to excite mTFP1 and mVenus fluorophores, which represent the donor and the acceptor, respectively. For proper image recording, hybrid detectors HyD were employed by gating a spectral acquisition window of 468–502 nm for the donor and 524–600 nm for the acceptor. In the photobleaching procedure, cells were bleached using the 514 nm argon laser beam at 100% intensity until the acceptor was photobleached down to about 10% of its initial value. FRET analysis was performed using ImageJ2, and the FRETcalc plug-in and FRET efficiency was calculated as previously described [38,39].

### 4.6. Statistical Analysis

Data analyses were performed using Prism statistical software (GraphPad, San Diego, CA, USA), and the values represent the mean ± SD obtained from at least three independent experiments. A normality test was performed using the D’Agostino & Pearson normality test. The statistical significance of the differences between two groups was determined using the two-tailed Student *t*-test for normally distributed values or the Mann–Whitney *U* test when the values were irregularly distributed. All *p*-values shown were two-tailed, and *p* < 0.05 was considered statistically significant.
